# Absence of post-treatment changes in sentinel lymph nodes does not translate into increased regional recurrence rate in initially node-positive breast cancer patients

**DOI:** 10.1007/s10549-023-07084-x

**Published:** 2023-09-07

**Authors:** Nina Pislar, Gorana Gasljevic, Ivica Ratosa, Anja Kovac, Janez Zgajnar, Andraz Perhavec

**Affiliations:** 1 Department of Surgical Oncology, Institute of Oncology Ljubljana, Zaloska 2, 1000 Ljubljana, Slovenia; 2 Faculty of Medicine, University of Ljubljana, Vrazov Trg 2, Ljubljana, Slovenia; 3 Department of Pathology, Institute of Oncology Ljubljana, Zaloska 2, Ljubljana, Slovenia; 4 Department of Radiotherapy, Institute of Oncology Ljubljana, Zaloska 2, Ljubljana, Slovenia; 5 Department of Medical Oncology, Institute of Oncology Ljubljana, Zaloska 2, Ljubljana, Slovenia

**Keywords:** Breast cancer, Sentinel lymph node biopsy, Neoadjuvant, Post-treatment changes, Regional recurrence

## Abstract

**Purpose:**

To determine whether the absence of post-treatment changes in the negative sentinel lymph nodes (SLN) in the neoadjuvant setting for biopsy-proven cN + disease results in an increased regional recurrence (RR) rate in patients after SLN biopsy (SLNB) only.

**Methods:**

Breast cancer patients with biopsy-proven cN + disease who converted to node-negative disease after neoadjuvant systemic treatment (NAST) and underwent SLNB only were included. Retrospective analysis was performed for patients diagnosed between 2008 and 2021. Pathohistological specimens were reviewed for the presence of post-treatment changes in the SLNs. Patients with negative SLNs (ypN0) were divided into two groups: (i) with post-treatment changes, (ii) without post-treatment changes. Patients’ characteristics were compared between groups. Crude RR rates were compared using the log-rank test. Recurrence-free (RFS) and overall survival (OS) for the entire cohort were calculated using Kaplan–Meier.

**Results:**

Of 437 patients with cN + disease, 95 underwent SLNB only. 82 were ypN0, 57 with post-treatment changes (group 1), 25 without post-treatment changes (group 2). During the median follow-up of 37 months (range 6–148), 1 isolated regional recurrence occurred in group 2 (RR rate 0% for group 1 vs. 4% for group 2, *p* = 0.149). There were no differences in 3-year RFS and OS between groups.

**Conclusion:**

Absent post-treatment changes in negative SLNs for biopsy-proven cN + disease that covert to node-negative after NAST did not result in increased regional recurrence rates in our cohort. Multidisciplinary input is essential to determine whether additional treatment is needed in these patients.

## Introduction

In breast cancer patients with clinically node-positive disease at presentation (cN +), axillary lymph node dissection (ALND) may be avoided after neoadjuvant systemic treatment (NAST) [1]. Three multicenter prospective studies have shown that sentinel lymph node (SLN) biopsy is feasible with an acceptably low false-negative rate (FNR) in patients who convert to clinically node-negative disease after NAST (ycN0) when three or more SLNs are removed [2–4].

If metastatic nodal infiltration was biopsy-proven prior to NAST, we expect to detect vital metastases or some degree of treatment effect in the removed SLNs [5]. Post-treatment changes are recognized as nodal fibrosis, calcifications, mucin pools, and foamy histiocyte aggregates on definite pathohistological examination [6]. If pathologic complete response (pCR) is achieved, post-treatment changes without residual metastases are observed, and if the response is partial, there are post-treatment changes with residual metastases [7]. In some patients with initial cN + disease, neither post-treatment changes nor metastases are detected in the SLNs after NAST, which may indicate that a false-negative lymph node was removed. If nodal pCR is recognized in SLNs, ALND can be omitted, whereas if residual metastases are detected, it is recommended to proceed with ALND [8]. If neither residual disease nor post-treatment changes are detected in SLNs, it is not clear whether ALND can be safely omitted. The aim of our study was to determine whether the absence of post-treatment changes in negative SLNs in biopsy-proven cN + disease results in an increased regional recurrence (RR) rate in patients after SLNB only. Secondary objective was to calculate recurrence-free survival (RFS) and overall survival (OS).

## Methods

After approval of the study by the National Ethics Committee (Approval Number 0120-178/2022/3), we retrospectively reviewed electronic patient records from the Institute of Oncology Ljubljana, Slovenia. We collected data from patients diagnosed with breast cancer and referred to our institution between January 2008 and December 2021, who received NAST followed by surgery. Female patients with cT1-4 cN1-3 tumors were included. Patients with bilateral or inflammatory carcinoma, a history of invasive/non-invasive breast cancer, synchronous cancer at other sites and patients who were pregnant or had distant metastases at presentation were excluded. Positive cN status was determined by axillary ultrasound (AUS), and lymph nodes that met the criteria for suspicious/positive underwent fine-needle aspiration biopsy [9]. Only biopsy-confirmed nodal metastases were considered cN +.

After NAST, axillary status was reevaluated by clinical palpation with/without AUS. Axillary surgery was planned according to national guidelines at that time; before the publication of the three prospective studies, patients with cN + disease rarely underwent SLNB and usually underwent ALND directly. In patients with cN + disease who converted to ycN0 after NAST, SLNB was planned after the practice was included in the national guidelines. At our institution, we perform SLNB after NAST in patients with initial cN + disease using the dual-tracer technique (technetium-labeled nano colloid and blue dye), without nodal clipping and remove three or more sentinel lymph nodes. We regularly use intraoperative touch imprint cytology (ITIC) and if the result is positive, we perform ALND during the same operation [10]. If lymphoscintigraphy is negative or we are unable to identify three or more SLNs, we proceed to ALND. Depending on definite pathohistological results, completion ALND is recommended if macro metastases are found in the SLNs. If SLNs contain isolated tumor cells (ITC) or micro metastases, the decision is made on a case-by-case basis in the multidisciplinary team (MDT) meeting. If SLNs are metastases-free, ALND can be omitted. If less than 3 SLNs are identified on definite pathohistology (inadequate SLNB), the decision to perform or omit completion ALND is made at the MDT meeting. Considering the initial clinical stage, radiotherapy (RT) of the nodal basins is recommended in the adjuvant setting, as well as adjuvant systemic treatment according to current guidelines.

For this retrospective analysis, hematoxylin & eosin (H&E) sections and immunohistochemistry (IHC) of excised lymph nodes were re-reviewed by a single pathologist with expertise in breast pathology for the presence/absence of post-treatment changes [6].

Patients who underwent SLNB only and were ypN0 (metastasis-free or with ITC) in the final pathohistological report were included in the present study. They were divided into two groups based on revised final pathohistological report: (i) ypN0 with post-treatment changes and (ii) ypN0 without post-treatment changes. Clinical and pathological characteristics were reported as median values with ranges for continuous variables and as absolute and relative frequencies for categorical variables. Baseline characteristics were compared between groups using the Mann–Whitney *U* test for continuous variables and the chi-square test for categorical variables.

Regional recurrence was defined as a recurrence in ipsilateral axillary, supraclavicular or intramammary nodal basins. Local recurrence was defined as ipsilateral breast or chest wall recurrence. Distant recurrence was defined as any evidence of distant metastasis. Time to recurrence was calculated from the date of surgery. Patients were censored at the time of event, death, or last follow-up, whichever occurred first. Crude RR rates were compared between groups using the log-rank test. Kaplan–Meier curves were constructed to estimate 3-year RFS and OS. RFS and OS were compared between groups using log-rank test. P values less than 0.05 were considered statistically significant. IBM SPSS Statistics for Windows (IBM Corp., Armonk, N.Y., USA) was used for the analysis.

## Results

**Fig. 1:**
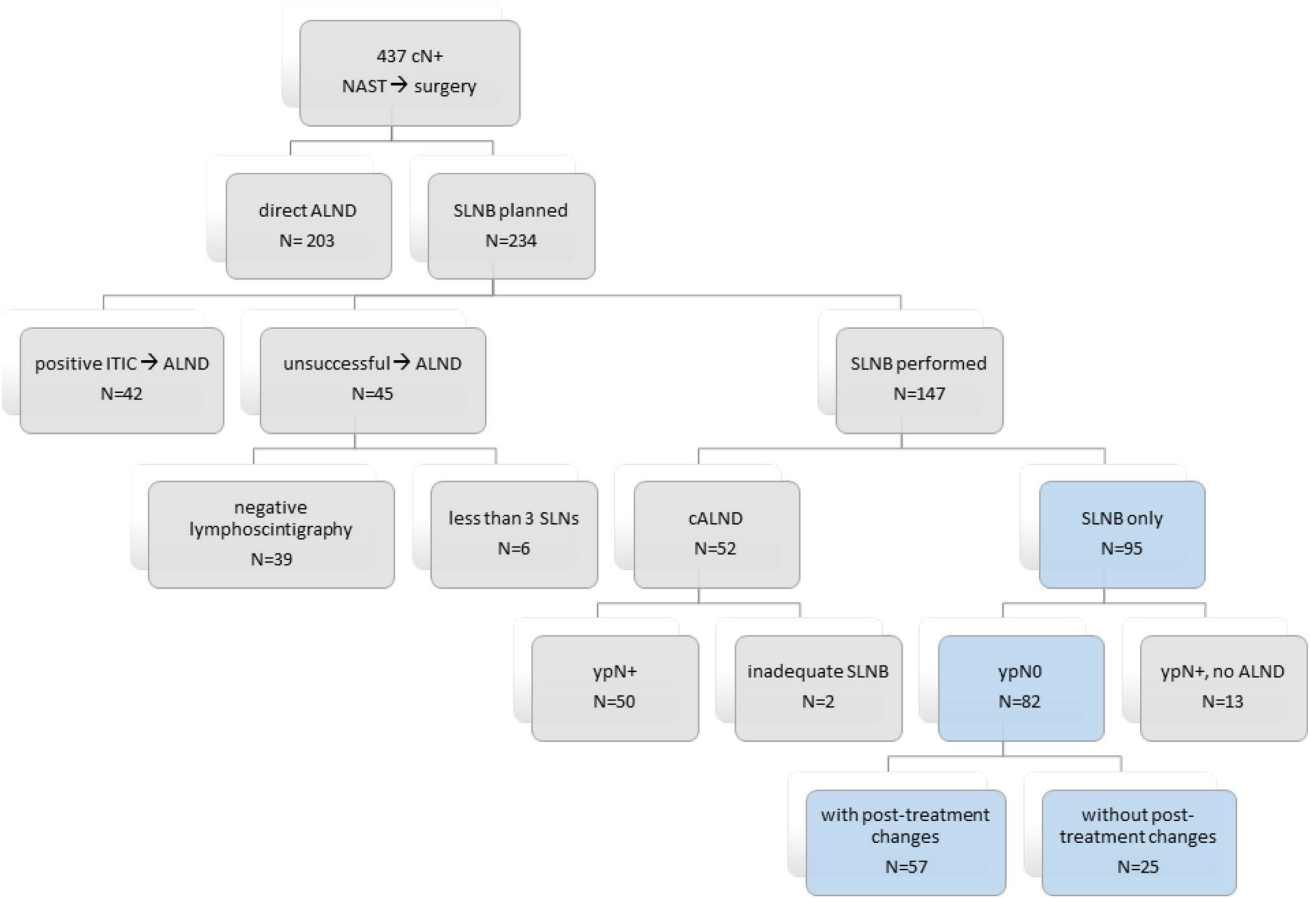
Flow chart of patient selection according to axillary procedure (cN +—clinically positive nodes before treatment, *NAST* neoadjuvant systemic treatment, *ALND* axillary lymph node dissection, *SLNB* sentinel lymph node biopsy, *ITIC* intraoperative touch imprint citology, *ypN* + positive nodes on pathology after neoadjuvant treatment, *ypN0* negative nodes on pathology after neoadjuvant treatment)

**Fig. 2:**
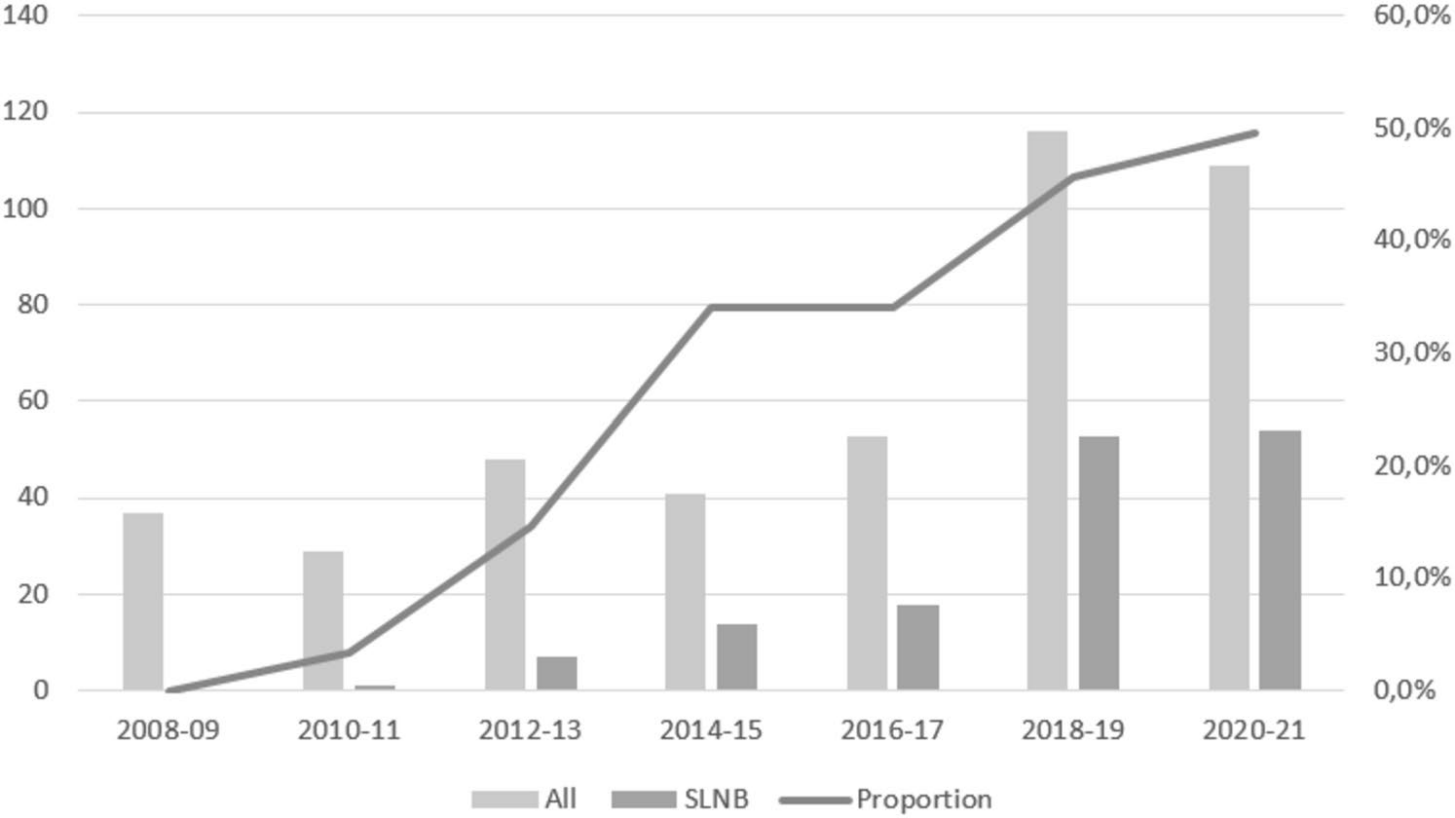
Time trend of axillary surgery for cN + →ycN0 patients after NAST between 2008 and 2021 according to primary surgery in axilla (*ALND* axillary lymphadenectomy, *SLNB* sentinel lymph node biopsy)

We identified 437 patients with cN + disease who underwent NAST followed by surgery. In 203, upfront ALND was performed. In the remaining 234 patients, SLNB was planned. Forty-two of them underwent completion ALND during the same surgery because of a positive ITIC result, 45 underwent ALND due to unsuccessful SLNB, and 52 underwent completion ALND as a separate procedure (because of metastases in the SLNs or inadequate SLNB). SLNB only was performed in 95 patients, 82 of whom were ypN0 and eligible for analysis. Figure 1 shows a flow diagram for patient selection, and Fig. 2 shows how the proportion of SLNB as primary surgery increases over time (*p* < 0.05).

After reevaluation of the H&E and IHC slides, we identified 57 ypN0 patients with post-treatment changes (group 1) and 25 ypN0 patients without post-treatment changes (group 2). Clinical and pathological characteristics between groups are compared in Table 1. Among 57 ypN0 patients in group 1, 10 patients also contained ITC. There were no patients with ITC among 25 in group 2. Based on the initial pathohistological reports previous to reevaluation, 52 ypN0 patients had evidence of post-treatment changes and 30 ypN0 patients had no evidence of post-treatment changes.

**Table 1:** Patients’ clinical, pathological and treatment characteristics

	ypN0 with post-treatment changes *N* = 57	ypN0 without post-treatment changes *N* = 25	*p*
Age in years, median (range)	50.1 (27–74)	46.4 (29–75)	0.248
Menopausal status			
Menopause	23 (74.2%)	8 (25.8%)	0.553
Pre/perimenopause	34 (68.0%)	16 (32.0%)	
Missing	1		
Tumor size at presentation in mm, median (range)	33 (12–80)	30 (15–60)	0.336
Nodal status at presentation (cN)			
cN1	41 (65.1%)	22 (34.9%)	0.282
cN2	10 (83.3%)	2 (16.7%)	
cN3	6 (85.7%)	1 (14.3%)	
Grade			
2	23 (71.9%)	9 (28.1%)	0.811
3	34 (69.4%)	15 (30.6%)	
Missing		1	
Histology			
Ductal	56 (70.0%)	24 (30.0%)	0.544
Lobular	1 (50.0%)	1 (50.0%)	
Subtype			
Luminal Her-2-	10 (62.5%)	6 (37.5%)	0.438
Her-2 +	36 (75.0%)	12 (25.0%)	
TN	11 (61.1%)	7 (38.9%)	
No. of SLNs retrieved, median (range)	3.9 (2–9)	3.0 (1–6)	0.007
No. of SLNs retrieved			0.014
2 or less	9 (60.0%)	6 (40.0%)	
3	9 (34.6%)	17 (65.4%)	
4	3 (12.0%)	22 (88.0%)	
5 or more	4 (25.0%)	12 (75.0%)	
Breast surgery			
BCS	29 (67.4%)	14 (32.6%)	0.984
Mastectomy	23 (67.6%)	11 (32.4%)	
Breast pCR (ypT0/Tis)	40 (76.9%)	14 (56.0%)	0.213
Received adjuvant RT	53 (93.0%)	24 (96.0%)	1.000
Regional recurrence	0 (0%)	1 (4.0%)	0.149

*Her-2* human epidermal growth factor receptor 2, *TN* triple negative, *SLN* sentinel lymph node, *BCS* breast conserving surgery, *pCR* pathologic complete response, *RT* radiation therapy

Among patients who underwent ALND during the study period and were ypN0, post-treatment changes were present in 88.9% (72/81).

We further examined the likelihood of finding post-treatment changes in SLNs according to the number of SLNs removed (Table 1).

### Regional recurrence

During a median follow-up of 41 months (range 5–149 months) 1/25 isolated regional recurrence was observed in group 2 (ypN0 without post-treatment changes). This represents 1.2% of the total cohort (1/82). RR rate was 0% for group 1 and 4% for group 2 (*p* = 0.149).

The patient underwent mastectomy with SLNB and had 3 SLNs removed at the initial surgery. All 3 SLNs were without post-treatment changes. She received adjuvant RT and tamoxifen. After 40 months, axillary nodal recurrence was confirmed. She underwent ALND with removal of 17 lymph nodes, 1 of which was positive. At 28 months after ALND, she remains disease-free.

### Recurrence-free survival and overall survival

**Fig. 3:**
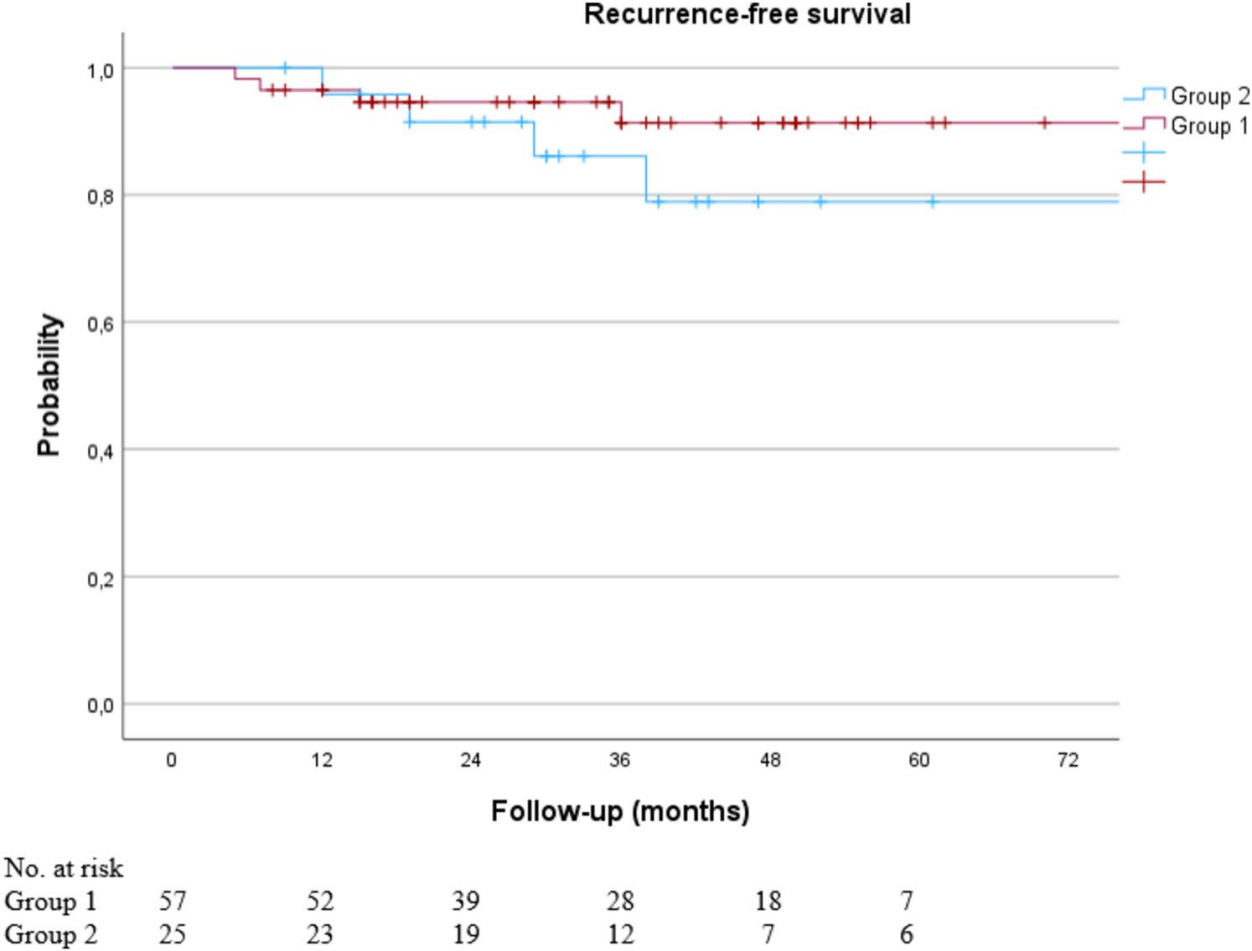
Kaplan–Meier curve of recurrence-free survival for ypN0 patients with and without post-treatment changes after sentinel node biopsy only

A total of 8 recurrences were observed (1 local, 1 regional, 6 distant). One patient had a local recurrence after breast-conserving surgery at 13 months. She had refused adjuvant RT and chemotherapy. Six patients suffered isolated distant recurrences. The 3-year RFS was 91.3% for group 1 and 86.1% for group 2, *p* = 0.255 (Fig. 3).

**Fig. 4:**
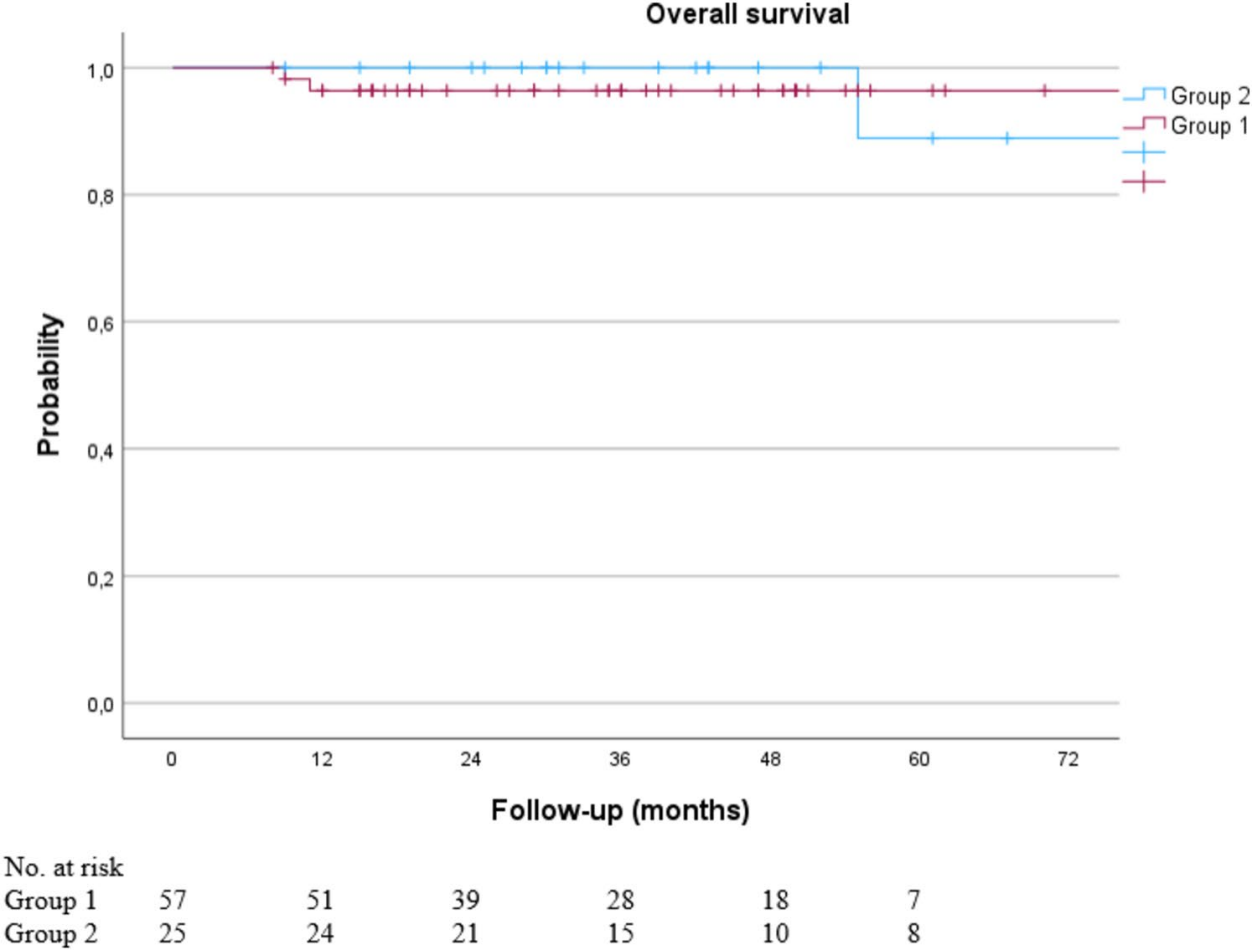
Kaplan–Meier curve of overall survival for ypN0 patients with and without post-treatment changes after sentinel node biopsy only

Three patients died during the median follow-up of 43 months (range 8–149 months). The 3-year OS was 96.4% for group 1 and 100.0% for group 2, *p* = 0.913 (Fig. 4).

## Discussion

Axillary management of initially node-positive patients who become node-negative after NAST remains controversial. However, with increasing evidence, the trend toward less invasive surgical procedures continues [11, 12]. Over the years, the number of breast cancer patients receiving NAST has increased at our institution. In cN + patients converting to ycN0 after NAST, we have adopted SLNB with double tracer technique and at least 3 SLNs removed. The proportion of upfront ALNDs at our institution has decreased accordingly (85% upfront ALNDs in 2012–2013, 61% in 2015–2016, and 50% in 2020–2021).

The main concern with SLNB in patients with cN + disease in the neoadjuvant setting remains the high FNR [13]. Although the purpose of axillary surgery is to both stage the axilla and remove any residual disease, it is unclear whether a higher FNR is actually associated with a higher regional recurrence rate and a worse prognosis. Detection of post-treatment changes in SLNs has been proposed as one of the principles for evaluating false negatives [6]. Brown et al. have shown that the absence of post-treatment changes in SLNs has a sensitivity of 82% and a specificity of 65% for detecting a false-negative SLN [14]. Post-treatment changes were present in 50% of SLNs, and the median number of SLNs removed was 2. In the study by Barrio et al., post-treatment changes were present in 88% of SLNs, and the median number of SLNs removed was 4 [15]. An alternative approach to assessing false negativity is clip placement at the time of initial nodal diagnostic biopsy. In the subgroup analysis of the Z1071 trial where a clip was used, it was identified in 75% of cases at SLNB only [16].

In our single-center retrospective study, post-treatment changes were identified in 70% of ypN0 patients (57/82) with a median of 3 SLNs removed. To our knowledge, we are the first to report the prognostic significance of absent post-treatment changes in SLNs in patients with biopsy-proven cN + disease undergoing NAST.

The probability of identifiyng post-treatment changes in our cohort of patients undergoing SLNB only was higher when more SLNs were removed, which supports the need for consistent sampling of at least 3 SLNs in cN + patients after NAST. However, the absence of post-treatment changes in SLNs did not translate into worse regional control in our cohort.

According to the literature, post-treatment changes were detected in more than 90% of ALND specimens [15]. This is comparable to our results; the patients that underwent ALND during our study period had post-treatment changes detected in 89%. In addition to false negativity, the absence of post-treatment changes in SLNs may also be explained by failure to identify the changes by the pathologist and to nodal sampling. In the present study, the first problem was partially adressed by reevaluation of the original H&E and IHC slides.

In our cohort of 82 patients with cN + disease who were ypN0 after SLNB only, there was only one (1.2%) isolated axillary recurrence during a median follow-up of 41 months. The regional recurrence rate in our cohort is within the range of previously published studies (0–1.6%) [11, 17–20]. The patient with regional recurrence underwent salvage ALND and is disease free 28 months after ALND.

Consistent with the study by Piltin et al., patients with ITC were classified as ypN0 in our study, although this is still controversial in the neoadjuvant setting [11]. Patients with ITC seem to carry a better prognosis than patients with macrometastases, but it is not entirely clear whether they can be classified as pCR/ypN0 [20–22]. The ongoing ICARO study may provide additional clarity on the oncologic outcomes for patients with ITC who undergo ALND, nodal RT or observation only after SLNB.

In women with cN + disease who respond well to NAST and are downstaged to ypN0, the role of RT in preventing locoregional recurrence is not entirely clear. The NSABP B-51 trial will shed light on the role of adjuvant RT in reducing recurrence rates in these patients. In our clinical practice, the need for regional nodal RT has been determined primarily by the status of the axillary nodes prior to NAST, regardless of response to treatment (ypN0 or ypN1). In our study, the vast majority of patients received adjuvant RT (93% of patients with post-treatment changes and 96% of patients whithout post-treatment changes received adjuvant RT).

Limitations of the current study include its retrospective nature and a median follow-up time of only 41 months. However, it has been shown that the majority of nodal recurrences occur in a follow-up period of up to 5 years, so a longer follow-up period would not likely change the results significantly [18, 23, 24]. Although we follow the national guidelines for performing SLNB after NAST in patients with cN + disease, which include the use of dual tracers and removal of at least 3 SLNs, we do not follow a very strict protocol but decide on a case-to-case basis whether to omit or complete ALND in patients with less than 3 SLNs removed or absent post-treatment changes. Multidisciplinary input is essential to decide whether additional treatment is needed in these patients.

The study allowed analysis of a single-center practice with many years of experience in the SLNB technique. Despite the relatively small number of patients included, this study adds to the short list of available studies on prognostic information for SLNB only after NAST in patients with cN + disease. To our knowledge, this is the first study to compare the oncologic outcomes of patients with cN + disease who convert to ycN0 and undergo SLNB only between patients with absent and present post-treatment changes in SLNs.

## Conclusion

In conclusion, absent post-treatment changes in negative SLNs in biopsy-proven node-positive disease converting to node-negative after NAST did not result in an increased regional recurrence rate in our cohort and should not be an indication for completion ALND in these patients. Longer follow-up is needed to further determine the oncologic safety of SLNB only in these patients.

## Data Availability

The datasets generated and analyzed during the current study are available from the corresponding author on reasonable request.
